# Exploring Empathy and Compassion Using Digital Narratives (the Learning to Care Project): Protocol for a Multiphase Mixed Methods Study

**DOI:** 10.2196/33525

**Published:** 2022-01-13

**Authors:** Manuela Ferrari, Sahar Fazeli, Claudia Mitchell, Jai Shah, Srividya N Iyer

**Affiliations:** 1 Department of Psychiatry McGill University Montreal, QC Canada; 2 Douglas Hospital Research Centre Douglas Mental Health University Institute Montreal, QC Canada; 3 Department of Integrated Studies in Education McGill University Montreal, QC Canada

**Keywords:** digital narratives, fundraising campaigns, mixed methods, randomized controlled trial, stigma and discrimination

## Abstract

**Background:**

Digital stories—first-person, self-made, 2- to 3-minute videos—generate awareness, impart knowledge, and promote understanding on topics such as mental illness. Digital stories are a narrative-based art form often created by individuals without formal training in filmmaking to relate personal experiences. Somewhat like digital narratives, video testimonies created within the social marketing or fundraising campaigns of government agencies and private or public corporations aim to reduce the stigma of mental illness while supporting research and services. In video testimonies, personal stories are captured on camera by professional filmmakers. Sharing critical life events greatly benefits tellers and listeners alike, supporting catharsis, healing, connectiveness, and citizenship.

**Objective:**

This study explores digital stories and video testimonies featuring mental illness and recovery in their ability to elicit empathy and compassion while reducing stigma among viewers.

**Methods:**

Using mixed methods, phase 1 will involve a search of Canadian social marketing activities and fundraising campaigns concerning mental illness and recovery. Phase 2 will involve the organization of digital storytelling workshops in which participants will create digital stories about their own experiences of mental illness and recovery. In phase 3, a pilot randomized controlled trial will be undertaken to compare marketing and fundraising campaigns with digital stories for their impact on viewers, whereas phase 4 will focus on knowledge dissemination.

**Results:**

Ethics approval for this study was received in March 2021. Data on the feasibility of the study design and the results of the controlled trial will be generated. This study will produce new knowledge on effective ways of promoting mental health awareness and decreasing stigma, with practical importance for future social marketing and fundraising campaigns. The anticipated time for completion within the 2-year study period includes 9 months for phase 1 (knowledge synthesis activities identifying social marketing and fundraising campaigns) and phase 2 (storytelling workshops), 11 months for phase 3 (feasibility assessment and data collection: randomized controlled trial), and 2 months for phase 4 (knowledge dissemination).

**Conclusions:**

The knowledge generated will have practical implications for the public and for future social marketing and fundraising campaigns promoted by government agencies as well as nonprofit and for-profit organizations by enhancing our understanding of how individuals and societies respond to stories of mental distress and what prompts citizens to help others.

**Trial Registration:**

ClinicalTrials.gov NCT04881084; https://clinicaltrials.gov/ct2/show/NCT04881084

**International Registered Report Identifier (IRRID):**

PRR1-10.2196/33525

## Introduction

### Background

Stories of human distress and struggle are shared daily through news stories and in social marketing and fundraising campaigns using digital formats, including videos [1,2]. Digital narratives are also created for public consumption by ordinary citizens with no formal training in filmmaking to promote awareness of different problems or realities [1]. Digital narratives and video testimonies aim to shape attitudes and stimulate empathy, compassion, and good citizenship among listeners [1,2]. Sharing moments and insights offered by personal experience is invaluable for tellers and listeners alike, promoting catharsis, healing, reconciliation, and social connection [3].

Researchers have studied the portrayal of mental health issues in various media (eg, television and newspapers) since the 1990s [4-7], identifying their impact as overwhelmingly negative. Media depictions abound with stereotypes of persons affected by mental illnesses as dangerous or violent, further contributing to their social marginalization and stigmatization [4-17]. In response, programs such as the Opening Minds campaign [18] have been created to encourage the dissemination of *good media reports* [19] that foster a more humanizing and hopeful understanding of the lived experience of mental illness and encourage help-seeking for mental health problems [20].

Although negative, media-generated portrayals of mental distress remain a serious cultural and social concern, new internet-based media forms offer a wider variety of perspectives [10,11], whether narratives created by government agencies and public or private corporations (video testimonies), or digital stories, a mix of images and text, and the visual arts of private citizens who use the internet to share personal experiences of mental illness as a way of promoting awareness [21,22]. Government or privately sponsored social marketing and fundraising campaigns use video testimonies to promote more positive perspectives, transforming the internet into an educational forum while eliciting financial contributions through organizational websites and social networking venues [23-28].

Preliminary evaluations report mixed results and raise many questions about the effectiveness of social marketing campaigns [29,30]. For example, the Cambridge intervention, which aimed to reduce discrimination against people with mental distress, succeeded in increasing public awareness of mental health issues through advertising to promote arts-based community interventions. However, the results were not sustained beyond the campaign, as has also occurred with other international initiatives [23]. Results of the Systematic Medical Appraisal, Referral, and Treatment Mental Health Project in India involving ≥2000 participants showed improved attitudes toward mental illness and reduced stigma around help seeking following a multimedia intervention that provided print information and a video on mental health [31]. The 2009 Opening Minds initiative of the Canadian Mental Health Commission also used media campaigns for public education on mental illness and stigma, with mixed results [26]. Moreover, the value of sharing recovery stories was not lost on those with lived experience who received training to perform a teaching role in the campaign [30,32]. Outcomes measured in terms of the attitudinal or behavioral intentions of listeners also showed success. The organizers agreed to create more focused, cost-effective, and sustainable grassroots public awareness campaigns following criticism that the Opening Minds campaign had not reached out to ethnic minorities or Indigenous people [29,30,33].

Another successful initiative, Bell Canada’s annual *Let’s Talk* antistigma fundraising campaign, takes on the stigma of mental illness while raising funds to support research and services. Video testimonies feature stories of celebrities such as Clara Hughes, a Canadian Olympic medalist who previously struggled with mental illness. In 2020, the campaign generated >154,173,435 *interactions* that contributed >CAD $7,900,000 (US $6,165,950) to mental health initiatives. Research showed a temporary increase in visits to mental health services among youth in Ontario, Canada, following the *Let’s Talk* campaign [34]. However, this campaign has been criticized for using testimonies that tended to downplay oppressive conditions such as poverty, racism, and violence connected with mental illness [35,36].

Digital storytelling, that is, first-person, self-made, 2- to 3-minute narratives prepared digitally and shared on the web [20,37,38], are viewed by their creators as an opportunity for communicating emotions and thoughts and destigmatize sensitive and marginalizing personal issues while bringing hope and encouragement to others [38]. Storytelling is an age-old tradition that brings people together through shared knowledge and experience [3,39]. Digital stories or short movies that combine personal narratives and images are modern versions of this tradition [32] and an art form that combines emotional charge, authenticity, and simplicity [39,40]. Leading this grassroots movement [40] is the Center for Digital Storytelling, established in the 1990s to promote the creation of digital stories by citizens, from secondary school students to seniors, including those with no knowledge of media production techniques [38].

Given the lack of research on the impact of social marketing and fundraising campaigns [20,41] and the creation and dissemination of digital stories about mental illness by people with no film background, this study examines these 2 storytelling forms (video testimonies presented in social marketing and fundraising campaigns vs digital stories) in terms of their ability to elicit empathy and compassion. Individuals with lived experience of mental illness will be invited to create and share digital stories exploring difficult, vulnerable, and meaningful moments in their lives with full awareness and acceptance of any painful thoughts and feelings they experience. This process will enable them to see their experiences as part of the human condition rather than as personal, isolated, or shameful events.

### Stories, Meanings, and Discourses

As Baldwin [3] describes, human beings are meaning-making creatures defined by and defining themselves through the stories they tell about themselves. Stories evoke emotions that can motivate changes in behaviors, attitudes, beliefs, and even the overall course of lives [3]. Stories bring together different realities, people, places, and times. Viewing stories from various epistemic and ontological standpoints reveals key elements: (1) stories are fluid, as people have many stories to tell and their experiential understandings change over time; (2) sharing stories fulfills the human need for connection; (3) listening to stories can be troubling, but listeners are not always ready to listen deeply; (4) how a life moment is recounted varies with individual experience; and (5) stories are generated by, and generate, discourse, inviting critical analysis [38,40]. Stories also use discourse voluntarily or involuntarily, and new discourses are generated in communicating experiences to others [42-44].

### Empathy and Compassion

Empathy, a key concept, has both cognitive and emotional domains. Cognitively, empathy requires an understanding of another person’s inner experiences, feelings, and perspectives regarding the outside world, whereas the affective domain involves entering the experiences and feelings of the other. The notion of *feeling with* another has been studied by phenomenologists contemplating how subject and object are enmeshed in prereflective existence [45]. Merleau-Ponty [46] described this as “the intertwining of my life with the lives of others, of my body with the visible things, the intersection of my perceptual field with that of others.” Empathy has been criticized from a theoretical standpoint, notably in *On the Problem of Empathy* by the philosopher Edith Stein [45], where she questions whether it is truly possible to feel what another person is feeling.

Compassion, from the Latin *compati,* means *to suffer with*. Compassion, described as the capacity to feel distress for, and desire to alleviate the distress of, another person cuts across theological, philosophical, and psychological traditions [46]. The Dalai Lama defined compassion as “a sensitivity to the suffering of self and others, with a deep commitment to try to relieve it” [46,47], whereas psychological models and therapeutic practices recognize the concepts of compassion and self-compassion [46-49]. The model of self-compassion by Neff [48,49], inspired by Theravada Buddhism, emphasizes cognitive over motivational elements and comprises (1) awareness and openness to distress; (2) kindness; and (3) capacity to share experiences of distress with others without shame, as an act of common humanity. Another example, compassion-focused therapy by Gilbert [50], develops (1) genuine motivation to care for self and others, (2) sensitivity to distress and needs, (3) the ability to connect with one’s pain to heal, and (4) tolerance toward distress in eliciting empathy and compassion.

### Aims and Research Questions

#### Overview

This study explores digital stories and video testimonies about mental illness, particularly those focused on mental health recovery, and how these stories elicit empathy and compassion. The following three questions will be addressed:

1. How are mental and emotional distress depicted in video testimonies presented in social marketing and fundraising campaigns versus digital stories made by people without formal training in filmmaking?
2. What impact does digital storytelling have on its creators, as people willing to revisit difficult life moments and transform them into digital videos?
3. How do digital narratives (video testimonies and digital stories) elicit empathy and compassion among viewers?

#### Qualitative Research Methodology

Qualitative research methodology will be used to investigate all study activities related to digital storytelling, based on the following three research questions:

1. What is the value of digital narratives (video testimonies presented in social marketing and fundraising campaigns) and digital stories created by individuals for retheorizing empathy and compassion?
2. How do digital narratives (video testimonies in social marketing and fundraising campaigns) influence empathy and compassion? What emotions emerge when people engage with these digital narratives as viewers?
3. How do digital stories created by ordinary people impact empathy and compassion? What emotions emerge when people engage with digital stories as viewers?

#### Quantitative Research Methodology

A pilot randomized controlled trial (RCT) will be performed as a feasibility study to assess the impact of video testimonies (social marketing and fundraising campaigns) versus digital stories.

The specific quantitative research question is as follows: are digital stories made by people without formal training in filmmaking more effective in eliciting empathy and compassion among viewers than the digital narratives used in social marketing and fundraising campaigns?

Using the Thabane et al [51] typology for feasibility studies, we also developed questions to test (1) process, (2) resources, (3) management, and (4) the scientific basis of our RCT. Examples of questions related to feasibility of the RCT include the following:

1. Process: what were recruitment rates and refusal rates for participation; how was randomization conducted?
2. Resources: how much time was required to conduct each stage of the protocol? How much time was required to recruit participants (university students)? How adequate was the web-based platform (REDCap [Research Electronic Data Capture]; Vanderbilt University) for collecting data?
3. Management: what was the research team’s capacity, expertise, and availability for completing the planned research activities? Were any important study variables omitted? Do the data show too much or too little variability?
4. Scientific basis: what were the reliability, validity, and trustworthiness of assessments used with the targeted population (university students) for this specific intervention? What was the estimated intervention effect? What was the estimated variance of the intervention effect?

The primary focus of this pilot study will be to gather data on the feasibility of the RCT; however, the following primary and secondary hypotheses have been generated (Textbox 1).

**Primary and secondary hypotheses.**

**Primary hypotheses**

- Change in empathy among participants using the Toronto Empathy Questionnaire [52]
- Change in compassion among participants using the Compassionate Love Scale [53]

**Secondary hypotheses**

- Change in positive emotions among participants using the Dispositional Positive Emotions Scale [54]
- Change in mental health self-stigma among participants using the Self-Stigma of Mental Illness Scale-Short Form [53]
- Change in public mental health stigma among participants using the Difference and Disdain Scales for Public Stigma [55]

## Methods

### Overview

**Figure 1:**
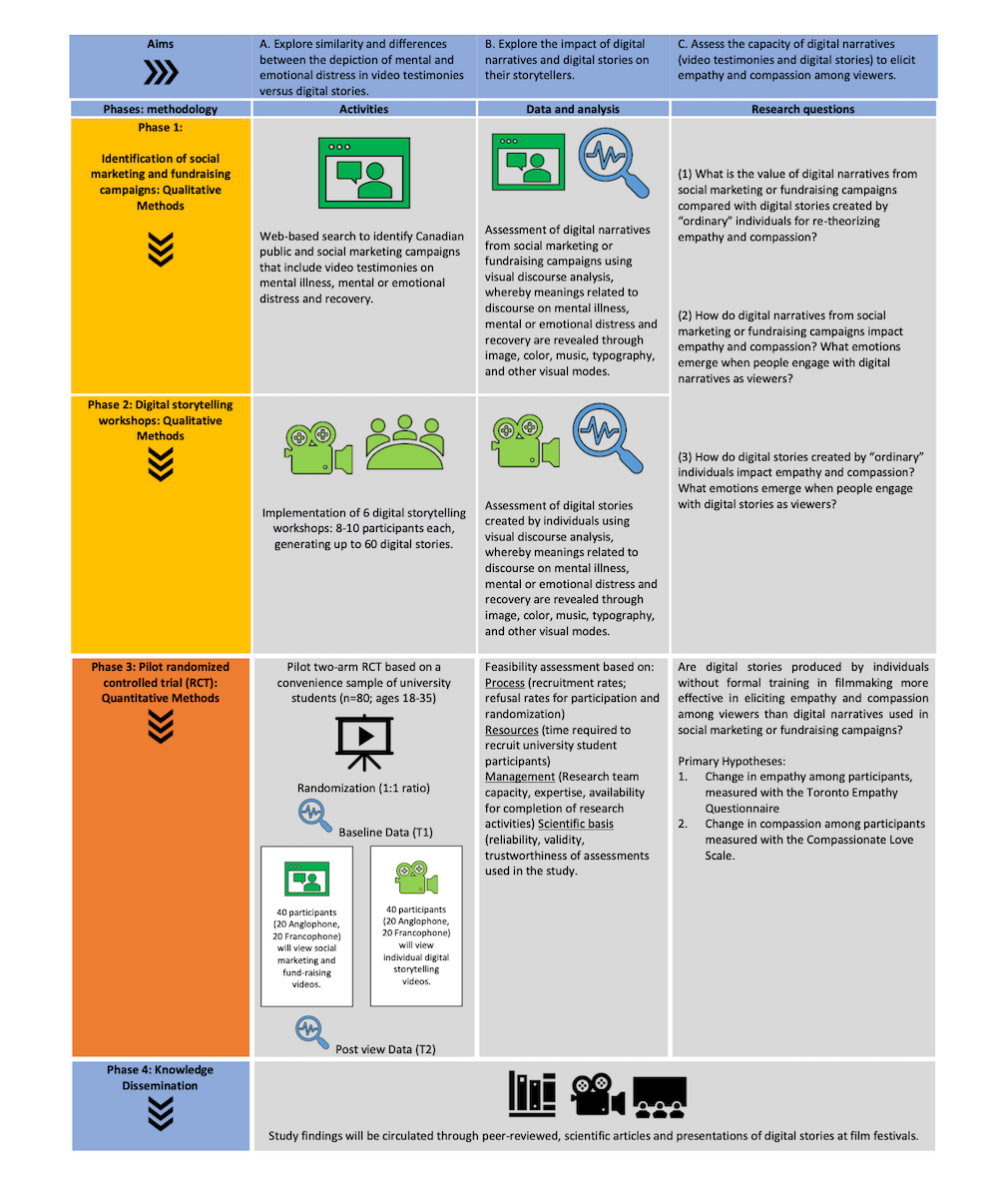
Learning to care project logic model.

This is a multiphase, mixed methods project that uses both qualitative and quantitative methodologies (Figure 1). Qualitative findings will be used to deepen the understanding of the quantitative results by (1) exploring differences between video testimonies and digital stories using discourse analysis and (2) conducting a thematic analysis of in-depth individual interviews. Phase 1 (months 1-9) will involve a preparatory search to identify Canadian social marketing and fundraising campaigns. Digital storytelling workshops (phase 2) will be held simultaneously, and in-depth interviews conducted before and after each workshop. In phase 3 (months 10-21), a pilot RCT will be undertaken, involving additional in-depth interviews. In phase 4 (months 22-24), knowledge mobilization will be conducted.

### The Study Process and Outline

#### Phase 1: Identification of Social Marketing and Fundraising Campaigns

##### Knowledge Synthesis Activities Identifying Social Marketing and Fundraising Campaigns (Completed)

Canadian public and social marketing campaigns that include video testimonies on mental illness, mental or emotional distress, and recovery were identified using a web-based search engine (Google). The websites of organizations dedicated to improving mental health and well-being (eg, the Canadian Mental Health Association, Canadian mental health hospitals, and their associated foundations) were identified and searched for campaign materials. All video testimonies identified from these campaigns and organizational websites were collected and will be analyzed. The search was supplemented by a Google video search to locate other publicly disseminated video testimonies. The searches were conducted from April to September 2021 to capture the most visible Canadian campaigns at the start of the study. Videos selected from each identified campaign included all the available first-person video testimonies, 1 to 5 minutes in duration, as well as television commercials and video advertisements in English and French. Campaign materials created as fact sheets involving substantial written text or image-based materials (eg, billboards, webpage advertisements, magazine advertisements, and slogans) were excluded. This study aims to gather 10 to 30 video testimonies for evaluation (phase 3).

##### Qualitative Discourse Analysis of Visual Materials and Interviews

The authors MF and SF will use visual discourse analysis [56,57] to assess the video testimonies (social marketing and fundraising campaigns) and digital stories created during the workshops (see the *Phase 2: Digital Storytelling Workshops* section). Discourse analysis appears to be an essential component in phases 1 and 2, which, in this phase, focuses on unpacking the meaning of texts, language, and other forms of communication in their social context [58]. Using this analytic method, visual data, video testimonies, and digital stories can be used to explore the construction of mental illness and recovery discourse in their digital forms [59]. In visual discourse analysis, image, color, music, typography, and other visual modes may be analyzed as languages [60-62], forming internally and externally coherent texts within the context in which they were produced [56]. Moreover, images and other visual modes may represent social relations between the producer, viewer, and represented object—an image vector of ideological positions [56]. The 3 perspectives from which visual content may be examined include contact (demand or offer), social distance (intimate, social, or impersonal), and attitude (involvement, detachment, viewer power, storyteller perspective on the story or video, equality, and representation power) [56]. The compositional meaning of images is analyzed through different systems: roles of the main character or protagonist and secondary characters in the video, information value (given or new, ideal or real, and important or less important), salience (achieved through size, color, and tone), and framing [56].

Following visual discourse analysis of the video testimonies (social marketing and fundraising campaigns) and digital stories, we will select the most suitable visual material for the RCT. A total of 2 homogeneous participant groups will be created to view the video testimonies and digital stories, respectively. The 2 groups will be similar in relation to illness experiences (eg, depression, anxiety, eating disorders, and psychosis) and language (English or French). The 3 dimensions for examining visual content (contact, social distance, and attitude) by Leeuwen [56] will be used as a guide to ensure similarity in the responses of the 2 groups regarding video testimonies and digital stories.

#### Phase 2: Digital Storytelling Workshops

##### Overview

MF will organize and facilitate the workshops. MF has previously implemented and evaluated a knowledge mobilization project using digital stories to explore care-seeking and access to care for psychosis. She will train 3 graduate students on digital storytelling practices. Workshop participants (aged 18-35 years) will be asked to create short, digital videos describing their subjective experiences of recovery from mental illness.

##### Recruitment

Social and community mental health services in Montreal and local universities will be contacted to promote, and host, 3-day digital storytelling workshops designed to produce a wide range of stories about recovery from mental illness that elicit emotions such as hope and compassion. Invitations to participate in the workshops will be posted (6 workshops: 8-10 participants each, generating up to 60 digital stories). Maximum variation sampling [63,64] will be used to determine participant composition in the workshops. Maximum variation sampling captures a wide range of perspectives by involving participants who represent a wide variety of identities, including gender, language, and sociocultural background, as well as different experiences of mental illness. Participants will be invited to an individual preworkshop meeting with the principal investigator where the subject matter for the stories (mental illness and recovery journey) and workshop structure will be explained.

Workshop participants (*storytellers*) will be invited to reflect on and depict moments involving emotions such as fear, shame, doubt, hope, courage, empathy, and warmth. Short digital videos created during each workshop will also be used for the evaluation of the study. The stories produced will remain the property of participants, who will each receive a media release form at the end of their workshop specifying if, how, and when their digital stories may be used. For this reason, we are soliciting the creation of approximately 60 digital stories for later use in the RCT. Workshop participants will be invited to a short interview before the workshop, and a longer interview soon afterward, to explore their experiences and story-sharing processes.

In-person workshops will be implemented based on participant availability and preferences. However, the workshops may also be implemented remotely for the duration of the COVID-19 pandemic to ensure participant safety. Specifically, participants will be offered a remote access option for involvement in the study (eg, preworkshop meeting with the principal investigator, screening, interviews, and data collection) using a secure, approved platform (Microsoft Teams). In addition, the web-based workshops will not operate under the full 3-day format, as originally planned, but will be split into short internet-based meetings, 3 hours each, and conducted over a 5-day period.

This project embraces *ethical practices in digital storytelling* [65]. The principal investigator and project coordinator will explain the consent form to participants at the preworkshop meeting, informing them of their rights, the purpose of the study, workshop structure and content, potential risks and benefits of sharing digital stories on the web, data collection processes and methods, and procedures for maintaining data confidentiality. Participants will have the opportunity to ask questions before signing the consent form. Written informed consent will be obtained from all the participants.

All workshop participants will participate in brief pre- and postworkshop interviews (n=60). Data will be analyzed thematically using a data-driven (inductive) approach rather than a theory-driven (deductive) approach. In this phase, the thematic analysis offers a useful method for examining the similarities and differences in the views of the research participants, generating rich insights. On the basis of a well-structured approach to handling data, thematic analysis is useful for producing clear, well-defined themes, subthemes, and relationships between them [55,66]. Following this approach, codes will reflect the discovered themes and meanings [67]. Braun and Clarke [66] guidelines for thematic analysis will be followed: (1) generating initial codes and searching for themes (collating codes into potential themes) and (2) reviewing and redefining themes.

#### Phase 3: Pilot RCT

##### Participants

A parallel 2-arm RCT design will be used to assess the impact of digital stories made by ordinary people compared with digital narratives used in social marketing and fundraising campaigns. A convenience sample of university students (n=80; age 18-35 years) from McGill University will be invited to participate in the pilot RCT, as a population comfortable with accessing web-based visual content and relatively easy to engage within the project timeline.

##### Sampling

The project coordinator will assess participant eligibility, obtain consent, and facilitate data collection (eg, log-in to the REDCap platform). Participants will choose an option for the screening and consent processes, whether in person, by phone, or video call using a secure platform (Microsoft Teams). Before seeking consent, the project coordinator will explain the consent form to RCT participants, informing them of the study objective, potential risks and benefits of participation, expected study duration, data collection processes, and methods and procedures for maintaining data confidentiality. Participants will be invited to ask questions before signing the consent form. Written informed consent will be obtained from all participants.

##### Randomization Procedure

Participants will be randomly assigned (1:1 ratio) to the social marketing or fundraising group (20/40, 50% Anglophone and 20/40, 50% Francophone participants) or the digital storytelling group (20/40, 50% Anglophone and 20/40, 50% Francophone participants) using the SAS, Stata, or R software. One of the arms of the pilot RCT will expose the participants to digital storytelling videos (intervention group), whereas the second arm will expose the participants to social marketing and fundraising videos (active control group). For both arms, sensitive content will be discussed with participants before screening of the videos so that certain content may be skipped over as needed.

The inclusion criteria for the study are as follows:

- Individuals of 18-35 years of age
- Individuals not currently treated in a hospital inpatient

The exclusion criteria for the study are as follows:

- Individuals outside the age range (18-35 years)
- Individuals currently treated in a hospital inpatient
- Individuals who attended a digital storytelling workshop and made a digital story used in the RCT

##### Methods of Assessment and Measurement

Participants will complete the first set of measures (time 1) before exposure to either study condition. Digital narratives will be assessed using a between-subjects design, in which each participant will view only video testimonies (social marketing or fundraising group) or only the digital storytelling videos (digital storytelling group). The system will record this information. Participants in each group will be exposed to a maximum of 10 video testimonies or digital stories. They will also complete standardized scales and a series of multiple-choice and open-ended questions regarding their perceptions of each digital video testimony or digital story. The duration of the survey will be 15 to 20 minutes.

Using a secure platform, a web-based survey comprising a variety of standardized scales, multiple-choice questions, and open-ended questions will be developed to assess video testimonies from social marketing or fundraising campaigns versus digital stories for their impact on empathy and compassion. The following scales will be used: Level of Familiarity scale (11-item questionnaire), which assesses the degree of contact with people living with specific diagnoses and will be used only before exposure to movies [68]; Toronto Empathy Questionnaire (16 items; Cronbach α=.79; test–retest reliability coefficient 0.73) [52]; Compassionate Love Scale (21 items; Cronbach α=.95; item-to-total correlations 0.46-0.81) [53]; Dispositional Positive Emotions Scale (5 items; Cronbach α=.80 [compassion subscale]; interscale correlation 0.44) [54]; Self-Stigma of Mental Illness Scale-Short Form (20 items; Cronbach α=.91) [69]; and Difference and Disdain Scales for Public Stigma (9 items) [70]. Examples of multiple-choice and open-ended questions are as follows:

- Why do you think this video was made? (response options: promote awareness, promote knowledge about services, promote knowledge about the organization, raise money, etc)
- How much control did the person in the video have over the story?
- How much control did the person in the video have over production of the video (eg, images music, etc)?
- How much control did the person in the video have over editing?
- How much control did the person in the video have over its dissemination or use?
- How much did watching these videos affect you?
- What was the most important thing you learned?

##### Data Management and Analysis

Mean group differences in scores on the selected measures from baseline to postintervention will be analyzed using repeated-measures analysis of variance, with intervention as the between-subjects factor and time the within-subjects factor, and with an interaction term (time and interaction group). To address feasibility questions, we will use descriptive statistics from standard operating procedures (eg, screening tools or data) and data collected (eg, number of participants, recruitment rates, and refusal rates).

#### Phase 4: Knowledge Dissemination

The knowledge dissemination plan will use targeted strategies and tools to make findings readily accessible. Identified knowledge users include academics from multiple disciplines, government policy makers, organizational decision-makers, and the public. Findings will reach the academic community through presentations at international conferences and publications in peer-reviewed journals. Web platforms will be used to reach government policy makers, nonprofit or for-profit organizations, and other stakeholders, favoring content in the form of brief reports (French and English) and infographics. With participant consent, digital stories will be submitted for release at future public events, including short film festivals, to promote ongoing public dialog.

### Trustworthiness, Validity, and Reliability

This study uses Lincoln and Guba [71] concept of trustworthiness, which is based on the following criteria: credibility, transferability, dependability, and confirmability. Credibility will be achieved using data collection triangulation and analysis. *Distinctive data collection activities will be implemented and a systematic search performed* to identify social marketing and fundraising campaigns (video testimonies) and analyzed using visual discourse analysis. Digital storyteller workshops will be implemented to generate digital stories that will be analyzed using visual discourse analysis. Interviews will be conducted with workshop participants to further investigate their experiences, and interviews will be analyzed using thematic analysis. At the end of each activity (data synthesis, implementation of workshops and interviews, and data analysis), the research team will hold a debriefing session on the research process, which will also increase credibility. Transferability, which refers to the generalizability of the qualitative inquiry and findings, will be operationalized through a detailed description of the methodological steps taken and an explanation of the process by which the definitive findings were generated and interpreted. To achieve dependability, the research team will document each phase so that the activities and decisions taken are transparent [71,72]. A logic model (Figure 1) describing the research process has been created to demonstrate dependability in the event of a study audit. According to Guba and Lincoln [73], confirmability, concerned with the grounding of interpretations and findings in the data, is established when credibility, transferability, and dependability are all achieved. As mentioned, the research team will rigorously disclose the reasons for the methodological and analytical choices made throughout the study [72].

To promote the validity and reliability of the RCT, this project has been registered, and CONSORT (Consolidated Standards of Reporting Trials) will be used to report the methodology and outcomes of the pilot trial.

## Results

The study was approved by the research ethics board of the Douglas Hospital Research Centre, Montreal, Canada, in March 2021. The study design adheres to the CONSORT guidelines for reporting pilot RCTs [74,75]. Anticipated time to completion within the 2-year study period includes 9 months for phases 1 (knowledge synthesis activities identifying social marketing and fundraising campaigns) and 2 (digital storytelling workshops), 11 months for phase 3 (feasibility assessment and data collection: RCT), and 2 months for phase 4 (knowledge dissemination).

The short- and long-term objectives of the project are to (1) analyze how mental and emotional distress is depicted in social marketing or fundraising campaigns versus digital stories created by *ordinary* people, (2) implement a digital storytelling workshop in the community and create a collection of digital stories that capture emotional encounters involving compassion (eg, fear, shame, doubt, hope, courage, warmth, and genuineness), (3) assess the impact of social marketing or fundraising campaigns and digital stories created by *ordinary* people for viewer empathy and compassion, (4) explore the subjective experience of creating a personal story about mental and emotional distress in relation to theoretical formulations of empathy and compassion, and (5) explore the subjective experience of viewing digital stories about mental and emotional distress and compare this experience with the theoretical formulation of empathy and compassion.

## Discussion

### Principal Findings

Portrayals of mental distress are a matter of cultural and social interest as new media products become available to the public. Studies published since the 1990s overwhelmingly conclude that formal media depictions are biased, promoting the stereotypical view of people who undergo distress emotionally as mentally ill, dangerous, violent, or insane. Various agencies, organizations, and corporations are actively working to provide alternative stories or narratives to those presented in mainstream media by using video testimonies in social marketing and fundraising campaigns and, ultimately, by taking advantage of the internet. The impact of this work is underresearched. However, preliminary evaluations of social marketing campaigns report mixed results and raise questions about their effectiveness. In addition, the first-person narrative, prepared digitally and shared on the web, provides an alternative to mainstream media stories.

People are increasingly using digital videos to share their stories, viewing this as an opportunity to understand their emotions and thoughts and come to terms with disgrace around sensitive personal issues and marginalization while providing hope and encouragement to others. The proposed study explores digital stories, particularly stories of mental illness and recovery, and their ability to elicit empathy, compassion, and sense of citizenship among viewers.

### Strengths and Limitations

This mixed methods study is the first to examine digital stories and video testimonies featuring mental illness and recovery in their ability to elicit empathy and compassion among viewers while reducing stigma.

The study will produce important knowledge on effective ways of promoting mental health awareness and decreasing stigma in the public, with practical application for future social marketing and fundraising campaigns.

Generalizability of the findings is limited to the Canadian and North American context, as the digital stories and video testimonies selected and evaluated in this study represent social marketing and fundraising campaigns in Canada or the testimonies of private citizens.

### Conclusions

People are increasingly using digital videos to share their stories, viewing this as an opportunity to understand their emotions and thoughts and come to terms with disgrace around sensitive personal issues and marginalization while providing hope and encouragement to others. The proposed study will explore digital narratives, particularly those dealing with mental illness and recovery, and their ability to elicit empathy, compassion, and a sense of citizenship among viewers. The knowledge generated will have practical implications for the public and for future social marketing and fundraising campaigns promoted by government agencies as well as nonprofit and for-profit organizations by enhancing our understanding of how individuals and societies respond to stories of mental distress and what prompts citizens to help others.

## Abbreviations

CONSORT: Consolidated Standards of Reporting Trials
RCT: randomized controlled trial
REDCap: Research Electronic Data Capture
